# A national, multicentre, randomised, controlled, parallel-arms, phase III clinical trial of neoadjuvant FOLFOXIRI and chemoradiotherapy versus neoadjuvant CAPOX/FOLFOX and chemoradiotherapy in patients with high-risk locally advanced rectal cancer: study protocol of the MEND-IT II trial

**DOI:** 10.1186/s12885-026-16072-5

**Published:** 2026-05-18

**Authors:** E. Banken, M. Borg, I. E. G. van Hellemond, S. G. van Ravensteijn, H. M. U. Peulen, A. E. Verrijssen, J. Nederend, A. W. Daniels-Gooszen, T. R. van Oudheusden, G. van Lijnschoten, J. G. Bloemen, H. J. T. Rutten, H. L. van Westreenen, J. W. B. de Groot, C. Verhoef, P. J. Tanis, B. A. Grotenhuis, A. G. J. Aalbers, I. M. Werter, M. D. den Hartogh, J. A. J. Douma, T. G. A. Calon, L. B. J. Valkenburg-van Iersel, G. L. Beets, J. B. Tuynman, M. P. W. Intven, S. M. J. van Kuijk, M. Berbée, T. E. Buffart, J. M. L. Roodhart, J. W. A. Burger, Dutch Colorectal Cancer Group

**Affiliations:** 1 Department of Medical Oncology, Catharina Hospital Eindhoven, Michelangelolaan 2, Eindhoven, 5623 EJ The Netherlands; 2 Department of Surgery, Catharina Hospital Eindhoven, Michelangelolaan 2, Eindhoven, 5623EJ the Netherlands; 3 GROW Research Institute for Oncology and Reproduction, Maastricht University, Maastricht, the Netherlands; 4 Department of Radiotherapy, Catharina Hospital, Eindhoven, the Netherlands; 5 Department of Radiology, Catharina Hospital, Eindhoven, the Netherlands; 6 Department of Pathology, PAMM Laboratory for Pathology and Medical Microbiology, Eindhoven, the Netherlands; 7 Department of Surgery, Isala, Zwolle, the Netherlands; 8 Department of Medical Oncology, Isala, Zwolle, the Netherlands; 9 Department of Surgery, Erasmus Medical Center, Rotterdam, the Netherlands; 10 Department of Surgery, Antoni Van Leeuwenhoek, Amsterdam, the Netherlands; 11 Department of Medical Oncology, Rijnstate, Arnhem, the Netherlands; 12 Department of Radiotherapy, Radiotherapiegroep, Arnhem, the Netherlands; 13 Department of Medical Oncology, Frisius Medical Center, Location Leeuwarden, Leeuwarden, the Netherlands; 14 Department of Internal Medicine, Division of Medical Oncology, Maastricht University Medical Center, Maastricht, the Netherlands; 15 Department of Surgery, Maastricht University Medical Center, Maastricht, the Netherlands; 16 Department of Surgery, Amsterdam University Medical Center, Amsterdam, the Netherlands; 17 Department of Radiotherapy, Utrecht University Medical Center, Utrecht, the Netherlands; 18 Department of Clinical Epidemiology and Medical Technology Assessment, Maastricht University Medical Center, Maastricht, the Netherlands; 19 Department of Radiation Oncology (Maastro), GROW School for Oncology and Reproduction, Maastricht University Medical Centre, Maastricht, the Netherlands; 20 Department of Medical Oncology, Amsterdam University Medical Center, Cancer Center Amsterdam, Amsterdam, the Netherlands; 21 Department of Medical Oncology, Utrecht University Medical Center, Utrecht University, Utrecht, the Netherlands

**Keywords:** Locally advanced rectal cancer, Total neoadjuvant therapy, Complete response, Organ preservation, Watch-and-wait approach

## Abstract

**Background:**

Total neoadjuvant therapy (TNT) has emerged as a promising treatment strategy for locally advanced rectal cancer (LARC), demonstrating higher pathological complete response (pCR) rates and enhanced disease-free survival. However, evidence specifically addressing high-risk LARC and the potential for organ-preserving approaches remains limited. The potential benefit of TNT may be further optimised when administered to patients with specific high-risk characteristics, associated with poor prognosis. The optimal TNT regimen for achieving favourable organ preservation and survival outcomes has not yet been established, as no studies have directly compared triplet-based induction chemotherapy with doublet-based TNT strategies. Consequently, there is considerable heterogeneity in the administration of chemotherapy within TNT regimens in clinical practice.

**Methods:**

This multicentre, parallel-arms, open-label, randomised, controlled, phase III trial will include 394 patients with non-metastasised, high-risk LARC, with WHO-performance status 0–1 and fit for FOLFOXIRI. High-risk LARC is defined as the presence of at least one of the following tumour characteristics: invasion of the mesorectal fascia (MRF) (i.e. T4b or evident invasion), extramural venous invasion (EMVI) grade IV, tumour deposits (TD), ≥ 2 enlarged lateral lymph nodes (LLN) (≥ 7 mm). Patients are informed about TNT versus chemoradiotherapy. Patients who will undergo TNT will be randomised 1:1 between 6 cycles of FOLFOXIRI, followed by chemoradiotherapy (25 × 2 or 28 × 1.8 Gy) or 4 cycles CAPOX/6 cycles FOLFOX, followed by chemoradiotherapy. Patients either undergo surgery or enter a watch-and-wait approach after neoadjuvant treatment. A watch-and-wait approach is considered in case of a clinical complete response (cCR), determined maximal 26 weeks after start of treatment. The primary outcome is complete response rate (i.e. pCR or sustained cCR at 1 year). The main secondary outcomes are disease-free survival, overall survival, regrowth rate, radicality, toxicity and completion rate of neoadjuvant treatment, treatment related toxicity, quality of life, and post-operative morbidity. Patients who are eligible and undergo neoadjuvant chemoradiotherapy, will be asked to participate in the observational cohort.

**Discussion:**

This protocol describes the MEND-IT II study, which compares complete response rates (pCR and cCR) between FOLFOXIRI-based and CAPOX/FOLFOX-based TNT in a homogeneous high-risk LARC population. The results of this study may contribute to optimise neoadjuvant treatment strategies and tailored treatment.

**Trial registration:**

Overview of Medical-Scientific Research in the Netherlands (OMON): NL-011486. Clinicaltrials.gov: NCT07472868.

**Supplementary Information:**

The online version contains supplementary material available at 10.1186/s12885-026-16072-5.

## Background

Neoadjuvant chemoradiotherapy has long been established as the standard of care for patients with locally advanced rectal cancer (LARC), with the primary aim of facilitating tumour downstaging and enhancing oncological outcomes. Despite the implementation of neoadjuvant chemoradiotherapy and advances in surgical techniques, LARC continues to be associated with high rates of recurrence and metastasis, with local recurrence observed in 5–10% of the patients and distant metastases occurring in 25–40% [1–3]. As a result, there has been increasing interest in intensifying neoadjuvant treatment with additional chemotherapy, a strategy known as total neoadjuvant therapy (TNT). TNT has been proposed to improve complete response rates, potentially allowing for more organ-preserving treatments, improved disease-free survival (DFS) and overall survival (OS) [4–6]. Currently, many patients have been treated with TNT internationally, with a variety of different regimens and without clear guidance or proof as to which regiment should be preferred.

The therapeutic impact of TNT may be further enhanced by improved patient selection, particularly focusing on patients with evident high-risk tumour characteristics within the LARC population. Several studies indicate that features as invasion of the mesorectal fascia, extramural venous invasion (EMVI), tumour deposits (TD), and enlarged extramesorectal lateral lymph nodes (LLNs) are associated with local recurrence and distant metastasis rates of up to 20 and 50% respectively, correlating with worse survival [7–17]. Patients with these high-risk characteristics may derive greater benefit from intensified neoadjuvant treatment. Firstly, MRF involvement is strongly linked to an increased risk of local recurrence (16.4% vs. 5.8%, *p* < 0.0001) and distant metastases (37.6% vs. 12.7%, *p* < 0.0001) when compared to tumours without MRF involvement [12]. Moreover, extensive MRF involvement (invasion ≥ 6 mm) correlates with worse survival outcomes compared to limited involvement (< 6 mm) [8]. A meta-analysis examining EMVI demonstrated an increase in the risk of both synchronous (OR = 5.68, 95% CI = 3.75–8.61, *p* < 0.0001) and metachronous distant metastases (OR = 3.91, 96% CI = 2.61–5.86, *p* < 0.001) [10]. Another study found a higher 5-year incidence of distant metastases in EMVI-positive patients, both with and without TD (46.9% and 36.7%, respectively), compared to EMVI-negative patients (24.8%, *p* = 0.0006) [11]. In addition, EMVI grade IV is associated with a higher risk of distant metastases (49% vs. 30%), as well as poorer disease-free survival (DFS) (42% vs. 55%) and OS (62 vs. 76%) compared to grade III [18]. The presence of TD in LARC is also linked to an increased risk of local recurrence and distant metastasis (6.3% and 38.9% vs 2.7% and 14.3%, respectively, for TD-positive vs. TD-negative tumours) [19]. Lastly, the Multicentre Lateral Node Study showed that pathological LLNs correlate with a 19.5% local recurrence rate and worse survival. [15]. Another Dutch Snapshot study found that patients with multiple enlarged lymph nodes had a higher 4-year lateral local recurrence rate compared to those with a single enlarged node (28% vs. 11%, *p* = 0.059) [20]. The concept of optimising patient selection to enhance the effectiveness of TNT was explored in a retrospective comparison between two Dutch tertiary referral centres. This study observed an improved complete response rate following TNT compared to neoadjuvant chemoradiotherapy (25.0% vs. 9.8%, *p* = 0.002) in patients with LARC with the presence of at least one high-risk criterium, and an improved 3-year OS compared to neoadjuvant chemoradiotherapy [21]. However, selection bias hampered the ability to draw definitive conclusions.

The available evidence on TNT is largely derived from four randomised controlled trials. The STELLAR trial, a multicentre phase III study, compared a TNT regimen with short-course radiotherapy followed by four cycles of CAPOX with standard chemoradiotherapy, and found a higher pathological complete response (pCR) or sustained clinical complete response (cCR) rate for patients treated with TNT (21.8% versus 12.5%, *p* = 0.002) [22]. While this study demonstrated an improved OS (86.5% versus 75.1%, *p* = 0.033) for patients treated with TNT, three-year DFS was comparable between both groups (64.5% versus 62.3%). In contrast, the POLISH II study, involving 515 patients with T3/T4 rectal cancer, found no clinically meaningful differences in pCR or OS rates when comparing short-course radiotherapy (5 × 5 Gy) followed by three cycles of FOLFOX with conventional chemoradiotherapy [23, 24]. The RAPIDO trial, which compared short-course radiotherapy (5 × 5 Gy) followed by six courses of CAPOX or nine courses of FOLFOX with neoadjuvant chemoradiotherapy, reported twofold higher pCR rates for the TNT group (28% vs. 14%, *p* < 0.001) [4]. Although this group had a lower risk of distant metastases (cumulative probability at 5 years 23.0% (95% CI, 19.2–26.8) vs. 30.4% (95% CI, 26.1–34.7), no difference in OS was observed, and these patients were more likely to develop local recurrence (10.2% versus 6.1%, *p* = 0.027) [5]. Lastly, the PRODIGE-23 trial evaluated a triplet-based TNT regimen (mFOLFIRINOX followed by chemoradiotherapy), comparing it with conventional neoadjuvant chemoradiotherapy, and also reported superior pCR rates for TNT (28% for TNT vs. 12% for chemoradiotherapy, *p* < 0.0001) [25]. Furthermore, they reported a survival benefit in favour of the TNT group, with 7-year OS rates of 81.9% vs. 76.1% in the chemoradiotherapy group (p = 0.033) [6]. The observed survival benefit may be attributed to the intensified triplet-based neoadjuvant therapy regimen administered in the TNT group. However, interpretation of the results requires caution, as it was based on a post-hoc analysis. The survival benefit became evident within the first year and remained consistent thereafter, although effects of chemotherapy typically become apparent after 18 months [26]. Consequently, the Dutch national guideline recommends considering TNT to increase pCR rates, improve DFS, and reduce the risk of distant metastases, but considers OS benefit by TNT insufficiently proven [27]. Within a TNT approach, systemic therapy may be administered using either doublet or triplet chemotherapy, as evidence demonstrating the superiority of triplet-based regimens over doublet regimens in patients with high-risk LARC is currently lacking. There are indications that triplet chemotherapy may be superior to doublet chemotherapy, particularly in the metastatic setting, where FOLFOXIRI plus bevacizumab improved progression-free survival (19.4 vs. 16.4 months, *p* = 0.0005) compared to FOLFOX plus bevacizumab [28].

All aforementioned studies have shared limitations, which we will briefly summarize. Firstly, organ preservation has not been adequately addressed, despite increased pCR rates following TNT and its potential to facilitate organ-preserving approaches. This gap is particularly relevant as surgical management of high-risk LARC often involves extensive procedures associated with considerable morbidity, functional impairment, and reduced quality of life [29–31]. A retrospective analysis by our study group revealed that 21.5% of the patients with high-risk LARC achieved a cCR after TNT and subsequently entered a watch-and-wait approach [32]. Another limitation is the heterogeneity of the study populations, where patients with high-risk LARC are underrepresented, potentially limiting the impact of TNT on long-term oncological outcomes in this group. Lastly, a variety of treatment regimens have been used across the conducted studies, making direct comparisons challenging. In fact, only one randomised trial has directly compared TNT with chemoradiotherapy, and studies comparing different TNT regimens are lacking. The lack of knowledge regarding the most appropriate TNT regimen, as well as the importance of appropriate patient selection for TNT calls for further studies focusing on patients with high-risk LARC, evaluating the effects of different TNT regimens.

In summary, TNT has emerged as promising treatment approach to improve response rates, organ-preserving treatments and oncological outcomes. Although current evidence remains inconsistent, TNT is increasingly implemented in clinical practice supported by results from the RAPIDO and PRODIGE-23 trial [4, 6]. However, careful consideration of the indication of triplet chemotherapy in a TNT regimen is warranted, given its association with higher treatment-related morbidity. Whether triplet chemotherapy is necessary for all patients with high-risk LARC to achieve optimal oncological and organ-preserving outcomes remains uncertain. The MEND-IT phase II trial, which selectively enrolled patients with high-risk LARC based on the presence of high-risk criteria explores the potential advantages of triplet TNT in this group [33]. Interim results of this study provided the rationale to support the power calculation of this study. The proposed study presents a phase III randomised controlled trial, aiming to compare the efficacy and toxicity of doublet-based versus triplet-based TNT in high-risk patients with LARC, providing crucial insights into the added value of triplet TNT and ultimately weighing the potential benefits against the risks of toxicity. These findings could impact LARC management, aiming to achieve a more tailored approach focused on organ preservation, quality of life, and oncological outcomes.

## Methods/design

### Study design and patient involvement

#### Study design

This is a multicentre, open-label, parallel-arms, randomised, controlled, phase III clinical trial. Patients will be informed about TNT versus chemoradiotherapy. All patients receiving TNT will be asked to participate in the study, and will be randomised to receive either neoadjuvant FOLFOXIRI, followed by chemoradiotherapy or neoadjuvant CAPOX/FOLFOX, followed by chemoradiotherapy. Both neoadjuvant treatments will be followed by surgery or a watch-and-wait approach. The primary aim of this study is to evaluate whether neoadjuvant FOLFOXIRI, followed by chemoradiotherapy, results in higher pCR or sustained cCR rate at 1 year compared to CAPOX/FOLFOX followed by chemoradiotherapy in patients with high-risk LARC. This study is planned to start in approximately 25 hospitals across the Netherlands.

Patients who meet the inclusion criteria but are not willing to participate in the randomised study and receive standard neoadjuvant chemoradiotherapy, are asked to participate in the observational (registration) cohort. Comparable data to those collected from the randomised patients will be collected to provide exploratory insights on outcomes.

#### Patient and public involvement

Patient participation has been integrated from the earliest stages of study development. The patient advocacy organisation “Stichting Darmkanker” and experienced patients from the “Adviesraad Endeldarmkanker” contributed by reviewing the study protocol and Patient Information Forms, ensuring that the patient perspective is well-represented and the information is clear and accessible.

### Participants, screening and randomisation

#### Eligibility criteria

Patients who are at least 18 years old, have histopathologically confirmed (and deemed) resectable high-risk LARC, with the lower edge of the tumour located at or below the sigmoidal take-off as assessed by a magnetic resonance imaging (MRI) scan, are eligible for inclusion. Additionally, patients must have a WHO performance status of 0–1 and be deemed fit for (dose-modified) triplet chemotherapy (FOLFOXIRI).

Exclusion criteria include an anticipated gross incomplete resection with visible tumour remaining post-surgery, tumour invasion into the neuroforamina, encasement of the ischiadic nerve, or invasion of the cortical area from S2 upwards, as these conditions are considered unresectable. Patients with distant metastases at the time of inclusion, homozygous dihydropyrimidine dehydrogenase (DPD) deficiency, recent chemotherapy (within the past 6 months), previous pelvic radiotherapy that interferes with the planned treatment, any contraindication to the planned therapy, or concurrent malignancies that would affect treatment or prognosis, or have mismatch repair deficient tumours (dMMR), will be excluded. Enlarged iliac or inguinal lymph nodes, as well as nonspecific lung nodules, are not criteria for exclusion.

#### Definition of high-risk LARC

**Fig. 1:**
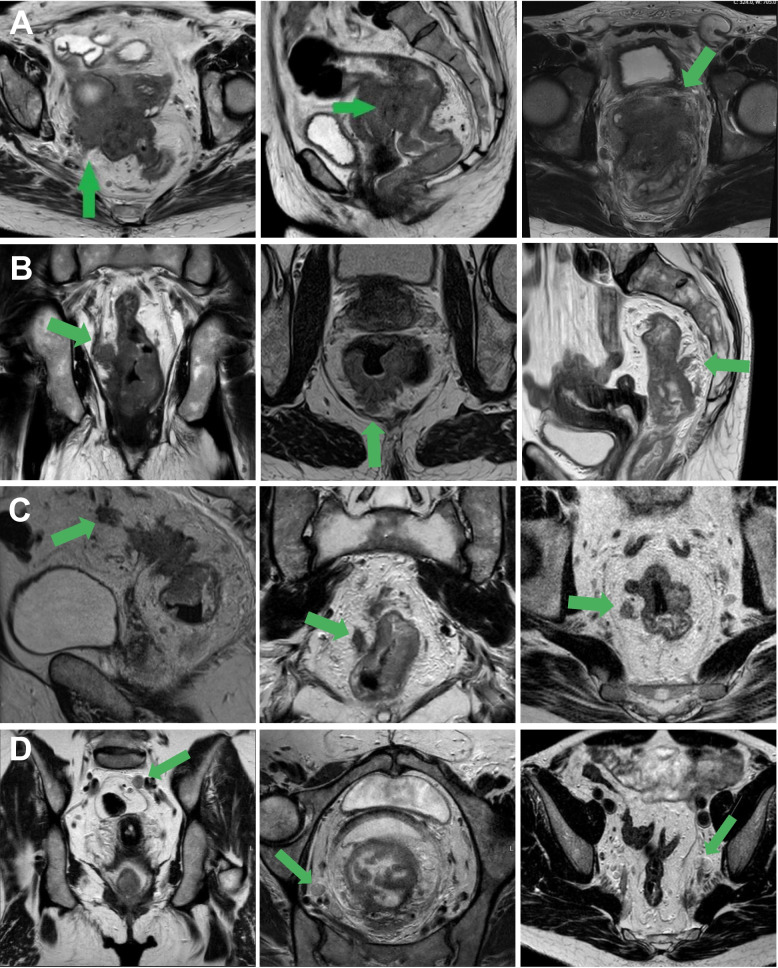
High-risk (‘’MEND’’) criteria. **A** Mesorectal fascia invasion; **B** Extramural Venous Invasion (grade IV); **C** Tumour deposits; **D** Lateral Lymph Nodes (≥ 7 mm)

To ensure that only patients who are proven to be at higher risk of treatment failure and poorer prognosis, and thus likely to benefit from TNT, are included in this study, high-risk criteria (MEND criteria, Fig. 1) have been identified and are applied for inclusion. High-risk LARC is defined by MRI-detectable tumour characteristics, including at least one of the following:

- The presence of a T4b tumour or evident tumour invasion of the MRF (distance to MRF 0 mm AND thickening of the MRF over a length of ≥ 5 mm);
- Grade IV extramural venous invasion (EMVI);
- Tumour deposits (TD);
- The presence ≥ 2 enlarged lateral lymph nodes (≥ 7 mm).

#### Screening

For inclusion, consensus on the eligibility of a patient should be reached during a multidisciplinary tumour board meeting (MDT). MRI-scans are assessed by a specialised radiologist in accordance with the Dutch and ESGAR guidelines [34, 35]. A radiological standardised operating procedure (SOP) and syllabus will be provided to all participating hospitals (supplementary files). A central MDT with a surgeon and radiologist is held once a month to discuss enrolled patients and to address any uncertainties regarding potential inclusions.

#### Recruitment and randomisation

All eligible patients with high-risk LARC presenting in one of the participating centres will be identified by their physician and discussed for eligibility during an MDT meeting. Randomisation will be performed by a central automatic randomisation tool (CASTOR) using block randomisation, with an arm A:arm B ratio of 1:1. Stratification will be performed per centre. Each patient is assigned a sequential subject number. The allocation is not blinded to the patient, the investigators or the treating physicians. This study will also include an observational cohort (registration arm) consisting of patients who meet the inclusion criteria but are not willing to participate in the randomised study, and opt for neoadjuvant chemoradiotherapy, followed by surgery or a watch-and-wait approach.

### Interventions

#### Neoadjuvant chemotherapy

All randomised patients will start with induction chemotherapy within 4 weeks after inclusion.

- ARM A, intervention (FOLFOXIRI):
  All patients randomised in arm A will receive induction chemotherapy with FOLFOXIRI, consisting of six two-weekly cycles. Dosages and method of administration follow the standard of care treatment protocols (dosages: irinotecan 165 mg/m2 body-surface area (BSA) intravenously (IV), oxaliplatin 85 mg/m2 BSA, leucovorin 400mg/m2 BSA, 5-fluorouracil 3200 mg/m2 BSA in 46 hours). Dose adjustments, including reductions, omissions, or delays, may be made based on toxicity assessments, at the discretion of the medical oncologist.
- ARM B, comparator (CAPOX/FOLFOX):
  All patients randomised in arm B will receive induction chemotherapy with CAPOX or FOLFOX. CAPOX consists of four three-weekly cycles and FOLFOX of six two-weekly cycles. Dosages and method of administration follow the standard of care treatment (dosages CAPOX: oxaliplatin 130mg/m2 BSA, capecitabine 1000mg/m2 BSA and FOLFOX: oxaliplatin 85mg/m2 BSA, leucovorin 400mg/m2 BSA, 5-fluorouracil 400mg/m2 BSA bolus and 2400mg/m2 BSA in 46 hours). Dose adjustments, including reductions, omissions, or delays, may be made based on toxicity assessments, at the discretion of the medical oncologist.

#### Treatment evaluation

After 4 cycles of FOLFOXIRI in arm A or 3 cycles of CAPOX/4 cycles of FOLFOX in arm B, local and distant restaging is performed using pelvic MRI and high-dose thoraco-abdominal computed tomography (CT) scan. If the disease is responsive or remains stable, a 5th and 6th cycle of FOLFOXIRI in arm A or 4th cycle of CAPOX/5th and 6th cycle of FOLFOX in arm B will be administered. In cases of distant metastases or when local disease becomes unresectable, best palliative care will be provided according to standard protocols. For patients with progressive local disease where surgery remains feasible, no further systemic therapy will be given, and treatment will proceed with chemoradiotherapy.

#### Neoadjuvant chemoradiotherapy

All randomised patients will start chemoradiotherapy within 6 weeks after the first day of the final induction chemotherapy cycle. Patients included in the observational cohort will start with chemoradiotherapy within 4 weeks after inclusion. Chemoradiotherapy will consist of 50 Gy of radiation administered in 2 Gy fractions, or 50.4 Gy in 1.8 Gy fractions (Intensity Modulated Radiotherapy (IMRT) or Volumetric Modulated Arc Therapy (VMAT) technique), with concomitant capecitabine (825 mg/m^2^ BSA) taken twice daily on radiotherapy days. Radiotherapy target volume delineation will be performed according to the national contouring guideline for rectal cancer (LPRGE consensus 2023).

#### Treatment evaluation

Restaging is performed 6–8 weeks after chemoradiotherapy using pelvic MRI and CT scan. MRI assessment follows the SOP, and all results are discussed in a specialised MDT meeting, which includes at least one surgeon, medical oncologist, radiologist and radiation oncologist. Treatment decisions are based on response, in consultation with the patient. For patients with a clear incomplete response and resectable disease, TME surgery is performed. In cases of good or near-cCR (≤ cT3, ycN0-1), endoscopy is conducted within 2 weeks following the MDT meeting, and a second response assessment is scheduled. A second local assessment, including pelvic MRI and endoscopy, is performed 14–16 weeks after chemoradiotherapy. For (near)cCR, a watch-and-wait approach is considered, with reassessment at 26 weeks. If an incomplete luminal response persists, TME surgery is performed, with local excision via TAMIS considered in selected patients. In cases of no further improvement of response, TME surgery is recommended. The third assessment, conducted at 26 weeks after chemoradiotherapy, includes pelvic MRI and endoscopy. TME surgery is performed in patients with a sustained incomplete response, while a watch-and-wait approach can be continued for patients with a cCR.

#### Watch and wait

For patients achieving cCR after neoadjuvant therapy, a watch-and-wait approach may be considered. A cCR is confirmed by imaging, endoscopy, and digital rectal examination. Patients are closely monitored according to standard care and local guidelines, with follow-up every 3 months in the first year, every 6 months in the second year, and every 6 to 12 months thereafter. Follow-up includes MRI and endoscopic evaluation, with biopsy performed if changes in the scar are detected.

#### Surgery

Surgery is performed by a surgeon experienced in rectal cancer procedures, typically within 10 to 14 weeks after completing chemoradiotherapy, following standard of care protocols. The surgical approach and extent are determined by the surgeon, with consultation from a reconstructive specialist if needed. The decision to include intraoperative radiation therapy (IORT) is at the surgeon's discretion.

#### Pathology

pCR should be determined by full evaluation of the tumour bed, embedding the tumour bed/scar with a maximum of 20 tissue blocks. All lymph nodes should be examined. For reporting the PALGA Protocol Module (PPM) should be used (standardised reporting PALGA).

#### Follow up and quality-of-life questionnaires

Follow-up will occur at 3, 6, 9, 12, 15, 18, 21, 24, 27, 30, 33, 36, 42, 48, 54, and 60 months postoperatively. At each visit, a blood sample will be collected to measure carcinoembryonic antigen (CEA) levels. If CEA levels increase, a high-dose thoracoabdominal CT scan will be performed per Dutch guidelines. Routine thoracoabdominal CT scans are scheduled at 6, 12, 18, 24, 30, 36, 48, and 60 months. Additional imaging may be performed as per local practice but is not required. For patients on the watch-and-wait approach, follow-up will follow standard care and local guidelines. All participants will complete validated quality-of-life questionnaires, including the QLQ-C30, QLQ-CR29, EQ-5D-5L, and QLQ-CIPN20 at baseline, after induction chemotherapy and chemoradiotherapy, and at 3-, 12-, and 24-months post-surgery. The WPAI-GH questionnaire will be completed at baseline, after induction chemotherapy, and after chemoradiotherapy. Questionnaires will be sent via mail or digitally, based on patient preference.

#### Participant timeline

**Fig. 2:**
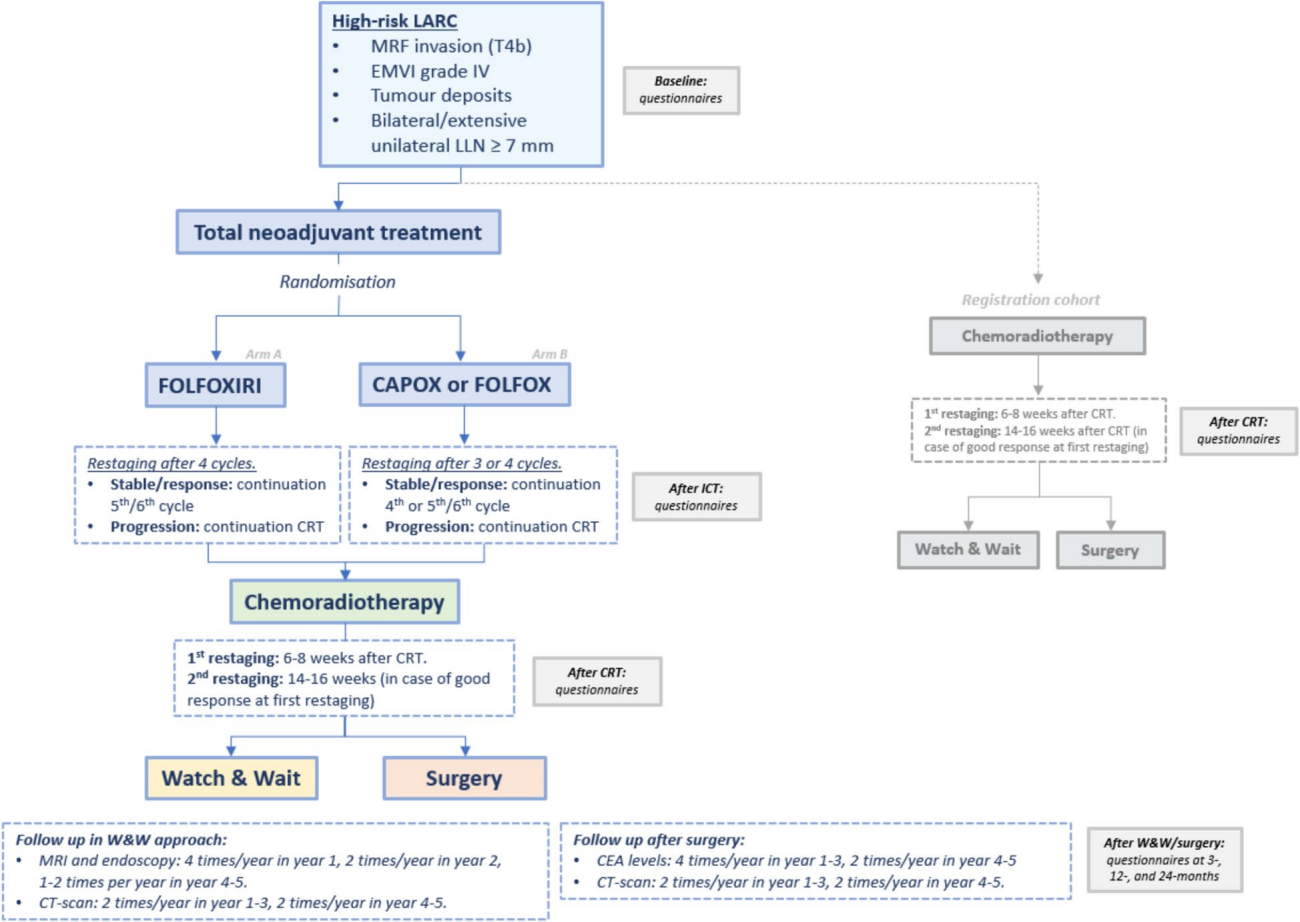
Study flowchart

A flowchart of the study and a timeline is presented in Fig. 2.

### Study outcomes

#### Primary outcomes

The primary outcome of this study is the complete response rate, defined as a pCR or a sustained cCR. A pCR is defined as the absence of residual tumour cells in the complete resected specimen including all resected regional lymph nodes (ypT0N0) according to the 8th edition of the UICC TNM classification of malignant tumours [36]. A sustained cCR is defined as the absence of viable tumour tissue based on MRI and endoscopic evaluation at 1 year after the last day of chemoradiotherapy.

#### Secondary outcomes

Secondary outcomes are regrowth rate, local recurrence rate, distant metastases rate, 3- and 5-years regrowth free survival (RFS), 3- and 5-years local recurrence free survival (LRFS), 3- and 5-years distant metastases free survival (DMFS), 3- and 5-years DFS, 3- and 5-years OS, successful organ preservation at 1 and 3 years, radiological response after induction chemotherapy, radiological response after chemoradiotherapy, toxicity of induction chemotherapy, toxicity of chemoradiotherapy, dose modifications and treatment delays during induction chemotherapy, dose reductions and treatment delays during chemoradiotherapy, completion rate of induction chemotherapy, completion rate of chemoradiotherapy, the number of patients undergoing surgery, surgical characteristics, pathological response, specimen quality, radicality, post-operative morbidity, quality of life, work-productivity, and cost-effectiveness and -utility.

All outcomes are also determined for the registration arm (observational cohort). These outcomes are intended solely to provide exploratory insights, as the study was not specifically powered to compare outcomes of this cohort.

### Data collection, management, and statistical analysis

#### Data collection and management

Central and local data management will be performed by Clinical Trial Centre Maastricht (CTCM). Data will be collected in an ISO 27001 certified study database (CASTOR) with an electronic case report form, according to the Good Clinical Practice guidelines and the Dutch legal requirements.

#### Statistical methods

All analyses will be performed according to the intention-to-treat principle. Relevant baseline characteristics, including age, sex, WHO performance status, and ASA score will be summarised using descriptive statistics. Additionally, details regarding the primary tumour are summarised using descriptive statistics, such as the type of neoadjuvant treatment, date and type of primary surgery, TNM-stage, tumour histology and surgical radicality. Categorical variables will be presented as frequencies and percentages. Continuous data will be summarised using descriptive statistics (mean, standard deviation, median, minimum, maximum, interquartile range). Statistical tests will be two-sided, with a *p*-value of < 0.05 considered statistically significant.

The proportion of patients achieving pCR and those who started a watch-and-wait approach with sustained cCR at 1 year will be reported as counts and percentages, stratified by treatment allocation. Mixed-effects (‘’multilevel’’) logistic regression will be used to compare arm A and arm B, with a random intercept at the centre level. Results will be expressed as odds ratios and 95% confidence intervals (CI).

Time-to-event endpoints (regrowth-free survival, LRFS, DFS, DMFS, and OS) will be visualised using the Kaplan–Meier method, with group comparisons performed using mixed-effects Cox proportional hazards regression. Results will be expressed as hazard ratio (HR) with 95% CI. Median survival times and 3- and 5-year survival probabilities, along with 95% CIs, will be reported, with survival rates provided if median times are not reached.

For datasets with ≥ 5% missing data, multiple imputation using fully conditional specification will be applied to complete the dataset. The number of imputations will be set to the percentage of incomplete records, and predictive mean matching will be used to draw imputations for continuous variables. Rubin’s rules will be applied to pool results across the imputed datasets for relevant analyses.

#### Sample size

The primary endpoint of this study is the complete response rate (pCR or cCR). Based on current literature, we expect a 32.5% complete response rate in the neoadjuvant triplet chemotherapy group, compared to 20% in the neoadjuvant doublet chemotherapy group. A 12.5% increase in response rate, with a 5% significance level and 80% power, requires a total sample size of 382 patients. Accounting for a 3% drop-out rate, the total required sample size is 394 patients, with 197 patients in each study arm.

The sample size calculation excludes the observational (registration) cohort, which will depend on patient enrolment during the trial's inclusion period. Data from this cohort will be used for exploratory purposes only.

### Safety and monitoring

#### Safety

The investigator shall report all serious adverse events (SAEs) and suspected unexpected serious adverse reactions (SUSARs) to the sponsor within 24 h of becoming aware of the event. The sponsor is responsible for reporting SUSARs to the European Medicines Agency (EMA) via the Eudravigilance database. Fatal or life-threatening SUSARs must be reported within 7 days, with a follow-up report due within 8 days. Non-fatal or non-life-threatening SUSARs must be reported within 15 days.

#### Data monitoring committee

To ensure patient safety throughout the study, a Data Safety Monitoring Board (DSMB) has been appointed to decide on the continuation of the study following the interim analysis. An interim analysis will be performed by the central data manager and the study statistician after 100 patients have entered a watch-and-wait approach or have undergone surgery. Inclusion will not be stopped at time of the interim analysis. The number of patients that cannot complete the full course chemoradiotherapy and the number of patients with major postoperative morbidity (Clavien-Dindo ≥ 3) will be tabulated and discussed with the DSMB. Looking at these safety and logistic aspects will have no consequences on the total sample size or the actual alpha level at final analysis. After the interim analyses, the DSMB will recommend continuation or discontinuation of the study.

#### Trial monitoring

The study will be monitored by a qualified monitor from the CTCM based on a predetermined monitoring plan.

#### Confidentiality

Patient information collected during this study will be managed in accordance with the Dutch General Data Protection Regulation (AVG). Additionally, the use of study numbers will preserve patient confidentiality.

## Discussion

The neoadjuvant treatment of patients with LARC has increasingly shifted towards TNT in recent years, in some cases triple-based, due to its proven higher rates of pCR, lower risk of distant metastases, and a potential effect on OS compared to chemoradiotherapy [5, 6, 21, 22, 25]. Nevertheless, the TNT approach remains a topic of debate, as previous studies have involved highly heterogeneous patient populations, which may have hindered the therapeutic impact of TNT on long-term oncological outcomes. Moreover, these studies have placed limited emphasis on evaluating organ-preserving strategies. The hypothesis exists that triplet-based neoadjuvant therapy may offer greater potential for organ-sparing treatments and improved long-term oncological outcomes compared to less intensive neoadjuvant strategies, particularly when patients are selected more accurately based on high-risk tumour characteristics. Therefore, the primary objective of this multicentre, parallel-arm, phase III study is to compare outcomes following a triple-based TNT regimen, consisting of FOLFOXIRI and chemoradiotherapy, with a less intensive doublet-based TNT regimen, consisting of CAPOX or FOLFOX and chemoradiotherapy, in a selected, homogeneous group of patients with non-metastasised LARC who meet at least one of the high-risk criteria ("MEND criteria"), associated with poorer prognosis.

This study seeks to determine whether the effectiveness of triplet TNT can be enhanced through improved patient selection based on well-defined high-risk tumour characteristics. Additionally, a key question for clinical practice is whether the potential oncological benefit of triplet-based TNT is clinically relevant enough to warrant the associated higher toxicity rates, or whether a doublet-based TNT regimen could suffice, offering potential advantages in terms of reduced toxicity and broader applicability for patients in daily practice. Moreover, although TNT has already been implemented internationally as the standard of care for patients with LARC, evidence supporting its superiority over chemoradiotherapy remains variable. Some authors have expressed criticism of TNT, citing a lack of convincing evidence for a clear survival benefit and questioning whether the available data can be reliably generalised across different healthcare systems [37]. Therefore, this study also includes an observational cohort to gain further insight into patients with high-risk LARC who will not undergo TNT and are treated with neoadjuvant chemoradiotherapy. Although the study is not powered for this comparison, the findings may contribute to a better understanding of the added value of triplet TNT compared with chemoradiotherapy alone.

In the context of this trial protocol, it may be questioned whether chemotherapy should be administered as induction or consolidation therapy, in light of the favourable outcomes regarding complete response rates and organ preservation following consolidation therapy observed in the OPRA and CAO/ARO/AIO-12 trials [38, 39]. However, the median interval from completion of chemoradiotherapy to restaging was 8 weeks in the induction chemotherapy arm, compared to 28.5 weeks in the consolidation chemotherapy arm [40]. A large cohort study showed that delaying surgery beyond 8 weeks after chemoradiotherapy increases the likelihood of achieving a pCR, suggesting that the induction chemotherapy group in the OPRA-trial may not have reached the full therapeutic effect at restaging [41]. It is hypothesised that induction chemotherapy may enhance tumour vascularity, thereby improving the efficacy of chemoradiotherapy and addressing early micro metastases. While induction chemotherapy may increase toxicity and postoperative complications, several phase II studies have reported manageable side effects and high compliance rates for subsequent chemoradiotherapy [42–44]. Moreover, administering chemoradiotherapy after induction chemotherapy extends the interval to surgery (waiting period of 10–14 weeks after chemoradiotherapy), which may allow patients more time to recover and will potentially enhance the downstaging effects of the chemoradiotherapy. Lastly, an enhanced effect of intraoperative radiotherapy (IORT), considered an additional treatment option for patients with high-risk LARC in the Netherlands, has been observed with a shorter time interval between preoperative (chemo)radiotherapy and IORT, though no evidence exits on outcomes after TNT in combination with IORT [45]. Given the potential benefits, administering neoadjuvant chemotherapy prior to chemoradiotherapy may be particularly advantageous for patients with high-risk LARC.

In summary, this is the first study to specifically focus on a high-risk subgroup within the LARC population, characterised by a poorer prognosis. This is a selective group of patients that has not been specifically addressed in previous studies, which have generally focused on broader LARC cohorts. Despite mixed evidence regarding the long-term benefits of TNT on oncological outcomes, TNT has been globally implemented as standard care for LARC patients, partly due to its positive impact on complete response rates and the potential for organ-preserving treatments. Triplet TNT may lead to improved oncological outcomes, particularly in high-risk LARC populations, but current literature lacks direct comparisons between TNT regimens. Moreover, it remains uncertain whether the potential oncological benefit justifies the risk of heightened toxicity associated with triplet-based TNT. This randomised controlled trial compares triplet-based TNT (FOLFOXIRI) with doublet-based TNT (CAPOX/FOLFOX) in a high-risk LARC population. The primary outcome, complete response rate, is of significant clinical relevance, particularly as it relates to organ-preserving strategies.Secondary outcomes will assess long-term oncological results. The results of this study are expected to contribute to more tailored treatment strategies, potentially enhancing both oncological outcomes and quality of life.

## Supplementary Information

Supplementary Material 1.

Supplementary Material 2.

## Data Availability

Data that support the findings of this study are available from the corresponding author upon reasonable request.

## Abbreviations

AE: Adverse events
cCR: Clinical complete response
CEA: Carcinoembryonic antigen
CT: Computed Tomography
CTCAE: Common Terminology Criteria for Adverse Events
DPD: Dihydropyrimidine dehydrogenase
DFS: Disease-free survival
DMFS: Distant metastases-free survival
DSMB: Data Safety Monitoring Board
EMA: European Medicines Agency
FDG-PET: Fluorodeoxyglucose Positron Emission Tomography
Gy: Gray
ICT: Induction chemotherapy
IORT: Intraoperative radiation therapy (IORT)
LARC: Locally advanced rectal cancer
LRFS: Local recurrence-free survival
dMMR: Mismatch repair deficient
MRI: Magnetic Resonance Imaging
OS: Overall survival
pCR: Pathological complete response
PPM: Palga protocol module
RFS: Recurrence free-survival
TNT: Total neoadjuvant therapy
SAE: Serious adverse events
SOP: Standard operating procedure
SUSAR: Suspected unexpected serious adverse reactions
