# Supplementary material for: A national, multicentre, randomised, controlled, parallel-arms, phase III clinical trial of neoadjuvant FOLFOXIRI and chemoradiotherapy versus neoadjuvant CAPOX/FOLFOX and chemoradiotherapy in patients with high-risk locally advanced rectal cancer: study protocol of the MEND-IT II trial
Source: BMC Cancer. 2026 May 18;26:838. doi: 10.1186/s12885-026-16072-5 (PMC13366820; doi:10.1186/s12885-026-16072-5)
Supplement: Supplementary file 2 — Supplementary Material 2. [file 12885_2026_16072_MOESM2_ESM.pdf]

MEND-IT II

Handleiding Beeldvorming

MRI

INHOUDSOPGAVE

1. Doel en toepassingsgebied van dit document 3
2. Algemene informatie 3
3. Beoordeling van de MRI 5
4. Contactgegevens 11

1. Doel en toepassingsgebied van dit document

Dit document is opgesteld ten behoeve van de MEND-IT II studie en dient als richtlijn voor het beoordelen van de lokale tumor op MRI's van patiënten met een hoog-risico lokaal gevorderd rectumcarcinoom die deelnemen aan deze studie.

Het doel van dit document is ervoor te zorgen dat alle deelnemende centra uniforme beoordelingscriteria hanteren voor de radiologische evaluatie.

2. Algemene informatie

Alle deelnemers ondergaan op 3 vastgestelde momenten gedurende de studie een MRI van het bekken: bij diagnose, na 3 of 4 kuren inductiechemotherapie (ICT) met FOLFOXIRI, CAPOX of FOLFOX en 6-8 weken na het beëindigen van de chemoradiotherapie (CRT). Het doel van de MRI is het in kaart brengen van de primaire tumor (inclusief de ongunstige kenmerken), het beoordelen van de resectabiliteit en het beoordelen van de respons gedurende neoadjuvante behandeling. Het continueren van de studiebehandeling is afhankelijk van deze beoordeling.

Door het toevoegen van inductiechemotherapie aan chemoradiotherapie gevolgd door chirurgie is een responsevaluatie met MRI noodzakelijk (tussen inductiechemotherapie en chemoradiotherapie). Mocht er sprake zijn van progressie van ziekte onder inductiechemotherapie , kan immers na 3 of 4 kuren overgegaan worden op CRT in plaats van het voltooien van de laatste (CAPOX) of 5^{e} en 6^{e} kuur (FOLFOXIRI en FOLFOX).

De MRI's zullen beoordeeld worden door radiologen met specifieke expertise in beeldvorming van het kleine bekken in de participerende centra.

Bij veel geïncludeerde patiënten betreft het een omvangrijke tumor, waardoor het essentieel is om een voldoende groot gezichtsveld (FOV) te hanteren, zowel in anteroposterieure richting als lateraal. Om dit te waarborgen kan overwogen worden om de transversale T2-sequentie niet te anguleren of aanvullend een extra niet-geanguleerde scan van het gehele bekken te maken. Dit is met name van belang voor de preoperatieve planning, omdat een niet-geanguleerde scan de operateur vaak een beter overzicht geeft van de uitbreiding van de tumor ten opzichte van omliggende structuren

De MRI scan dient minimaal te voldoen aan de eisen gesteld in de Nederlandse richtlijn colorectaal carcinoom, hieronder samengevat:

- 1.5 Tesla (T) of 3T MRI scanner.

- 'Phased-array receiver coil' voor beeldvorming van het kleine bekken.
- T2-gewogen hoge resolutie in minimaal 3 richtingen, een coupe dikte van maximaal 3mm en de afwezigheid van vet onderdrukking.
- Diffusie gewogen beeldvorming (DWI): minimaal b-waarde ≥ b800 en 'ADC (apparent diffusion coefficient) maps'.
- Het gezichtsveld (FOV) van zowel de T2 als de DWI moeten het volledige tumorgebied beslaan.

De volgende aanvullingen zijn optioneel, maar niet verplicht:

- Niet geanguleerde transversale T2, van het gehele bekken.
- T1 gewogen sequenties
- Vet onderdrukking
- Dynamische postcontrast sequenties
- Patiënt voorbereidingen (b.v. spasmolytica of micro-klysma)

3. Beoordeling van de MRI

1. MRI bij diagnose

Bij de beoordeling van de MRI dienen ten minste de volgende stappen doorlopen en genoteerd te worden in de verslaglegging. Geadviseerd wordt om dit op te nemen in een standaard-verslag format. Template voor Sectra-PACS is op aanvraag beschikbaar.

**Stap**	**Actie**
1	Algemene beoordeling van de tumor:<br>- Maximale diameter tumor (in mm)<br>- Type tumor (solide, mucineus, in combination with fistula/abcess)<br>- Aantal cm vanaf de anorectale overgang<br>- Relatie tot de sigmoid take-off: benoem tumoren waarvan de onderrand op of distaal van de sigmoid take-off is gelegen als rectumcarcinoom.<br>- Maak een tweedeling in lokalisatie:<br>o Proximaal rectumcarcinoom: onderrand van de tumor meer dan 5 cm afstand van de anorectale overgang en distaal van of op het niveau van de sigmoid take-off;<br>o Distaal rectumcarcinoom: onderrand van de tumor op of minder dan 5 cm afstand van de anorectale overgang.
2	Beoordeel het T stadium:<br>- T0: geen primaire tumor te identificeren.<br>- T1: tumorinvasie in de submucosa<br>- T2: tumorinvasie in de muscularis propria<br>- T3: tumorinvasie in de subserosa of in het peri-rectaal weefsel, zonder invasie in overige organen<br>o 3a: <1mm voorbij de muscularis propria<br>o 3b: 1-5mm voorbij de muscularis propria<br>o 3c: 5-15mm voorbij de muscularis propria<br>o 3d: ≥15mm voorbij de muscularis propria<br>- T4: tumorinvasie in andere organen/structuren en/of perforatie van het viscerale peritoneum<br>o 4a: tumor penetreert het oppervlak van het viscerale peritoneum<br>o 4b: tumorinvasie of verbinding met andere organen/structuren: geen, vagina, uterus, prostaat, vesiculae seminalis, blaas, urethra, darm, ovaria, peritoneum, ureter, S1, S2, S3, S4, S5, os coccyx, presacrale fascie, presacraal periost, os sacrum, levator ani, perineum, laterale bekkenwand, interne anale sfincter, externe anale sfincter, puborectalis, piriformis, obturator, ischiococcygeus, anders namelijk: …<br>NB. Noem peritoneale invasie ter plaatse van de omslagplooi cT4a. Stadieer alleen directe invasie als cT4a; contact van de rectumwand zonder tumordoorgroei met de omslagplooi is niet voldoende om van invasie te spreken.
3	Beoordeel het N stadium conform meest recente richtlijn:<br>- Zeker cN0: benoem klieren <5 mm korte-as als benigne, tenzij mucineus.<br>- Twijfelachtig cN+: beschrijf als twijfelachtig, te stadiëren als cN0.<br>- Zeker N+: benoem mucineus en korte-as >9 mm altijd als verdacht. Noteer aantallen verdachte lymfeklieren en tumordeposities.

	Wees beducht op overstadiëring en kies bij twijfel het laagste stadium.
4	<u>Beoordeling betrokkenheid mesorectale fascie (MRF):</u><br>Beoordeel de betrokkenheid van de MRF conform de meest recente richtlijn: Classificeer de MRF als betrokken (MRF+) wanneer EMVI, tumor deposities of irregulair begrensde klieren ≤ 1 mm afstand hebben tot de MRF. Beschrijf dit separaat.<br><br>Geef hierbij een extra categorie naast het gebruikelijke onderscheid in niet betrokken, wel betrokken (afstand ≤ 1 mm):<br>o Niet betrokken<br>o Wel betrokken, geen MEND-IT criterium<br>o Wel betrokken MEND-IT +*<br>o Wel betrokken, T4b.<br><br>* substantiële betrokkenheid, gedefinieerd als betrokkenheid van minimaal 5 mm in diameter (willekeurig vlak).
5	<u>Beoordeling aanwezigheid EMVI:</u><br>**Negatief:**<br>- Graad 0: het patroon van tumor uitbreiding door de spieren is niet nodulair en er is geen sprake van aangrenzende vaten.<br>- Graad 1: minimale extramurale stranding of nodulaire uitbreiding, echter niet in de nabijheid van een vasculaire structuur.<br>- Graad 2: Stranding in de nabijheid van extramurale vaten, echter met een normaal aspect en zonder duidelijk tumorsignaal in het vat.<br>**Positief, MEND-IT –:**<br>- Graad 3: Matige signaalintensiteit zichtbaar in de vaten, echter met een slechts lichte verandering van de contour en het aspect van de vaten.<br>**Positief, MEND-IT +:**<br>- Graad 4: Duidelijke irregulaire vaatcontour of nodale uitbreiding van het vat met duidelijk tumorsignaal<br>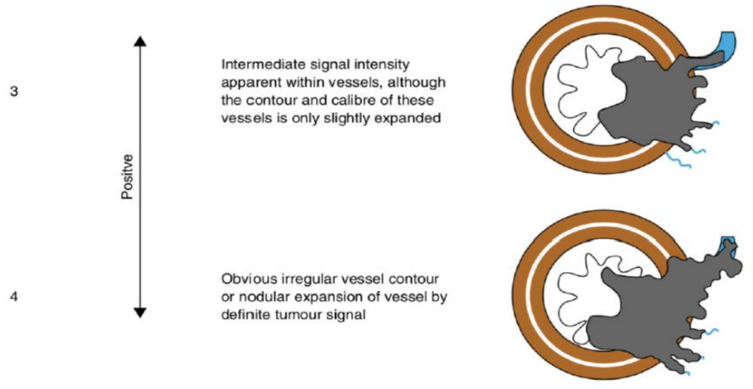
6	<u>Beoordeling aanwezigheid tumor deposities (TD):</u><br>- Geen (restant) normale lymfklier herkenbaar<br>- Irregulaire begrenzing<br>- In het beloop van vaten en onderbroken vaten.<br>- Uitbreiding in vaatstructuur (zogenoemde 'vascular tail').<br>- Bevindt zich los van de primaire tumor.

	Benoem bij zichtbare tumordeposities aantal en locatie, en classificeer dit tevens als cN+.
7	<u>Beoordeling aanwezigheid extramesorectale (laterale) lymfeklieren:</u><br>- Aantal zichtbare vergrote (≥ 7 mm) laterale lymfeklieren, of mucineuze klieren<br>- Beschrijving compartiment van zichtbare laterale klieren: iliacus internus, iliacus externus of het obturator compartiment<br>- Korte-as lengte in aantal mm (inclusief cortex) van de laterale klieren op een axiale, sagittale of coronale afbeelding.<br>- **MEND-criterium:** Aanwezigheid van ≥ 2 vergrote laterale lymfeklieren (LLN) met een korte-as lengte ≥ 7mm
8	<u>Voorstel tot radiologisch verslag primaire stadiering inclusief aan- of afwezigheid MEND-criteria:</u><br>Voorstel tot stadiering:<br>T 0 | 1 | 2 | 3a | 3b | 3ab | 3c | 3d | 4a | 4b N 0 | +, MRF - | +;beperkt | +;MEND-IT, EMVI - | + (graad 3) | + (graad 4), tumordeposities aanwezig | afwezig, geen verdachte laterale klieren | verdachte laterale klier rechts | verdachte laterale klier links | verdachte laterale klieren beiderzijds.

Voorstel voor verslag primaire stadiering rectumcarcinoom:
Aspect tumor: annulair | semicirculair | ulcerend | poliepeus | schotelvormig.
Morfologie: solide | mucineus.
Rand: infiltrerend | glad.
Tumor reikt tot in: mucosa | submucosa | muscularis | pararectaal vet | peritoneale omslagplooi | mesorectale fascie.
Invasieve component: van [XX] uur tot [XX] uur, maximaal [XX] mm groei buiten de wand.
Distale grens op: [aantal] mm vanaf de anorectale overgang (puborectale sling).
Proximale grens op: [aantal] mm [onder | boven] de peritoneale reflectie.
Betrokkenheid sigmoid-take-off: Zowel boven als onder | Volledig boven (sigmoidcarcinoom) | Volledig onder
Lengte tumor: [aantal] mm

MRF: niet betrokken | betrokken, beperkt | betrokken, MEND-IT + (invasie)].
Kortste afstand tumor tot MRF: [aantal] mm op [aantal] uur
MRF betrokken over [XX] mm
Uitbreiding buiten TME vlak: nee | ja

T-Stadium: T1 | T2 | T1/T2 | T3a | T3b | T3ab | T3c | T3d |T4 (visceraal) | T4 (peritoneaal)

Extramurale veneuze invasie:
Geen | gering/mogelijk: graad 3 | zeker aanwezig: graad 4.
Betrokken vat: V. Rectalis inferior | media | superior.

Klieren:

Geen | 1 | 2 | 3 | 4 | 5 | 6 | 7 | 8 | 9 | >9 verdachte lymfklieren.
Vasculaire deposities: nee | ja | mogelijk.
Verdachte laterale klieren: nee | links | rechts | beiderzijds.

N-Stadium: N0 | N+, met | zonder tumordeposities

Conclusie:
[conclusie]

Voorstel tot stadiering:
T 0 | 1 | 2 | 3a | 3b | 3ab | 3c | 3d | 4a | 4b N 0 | +, MRF - | +;beperkt | +;MEND-IT, EMVI - | + (graad 3) | + (graad 4), tumordeposities aanwezig | afwezig, geen verdachte laterale klieren | verdachte laterale klier rechts | verdachte laterale klier links | verdachte laterale klieren beiderzijds.

2. Restadiëring MRI na ICT en CRT

**Stap**	**Actie**
1	Beoordeling conform stappenplan genoemd hierboven (MRI bij diagnose).<br>- Herhaal stap 2 en 3 van MRI bij diagnose.<br>- Noem ycT stadium<br>- Noem ycN stadium: geef aantal verdachte klieren én aantal verdachte tumordeposities apart.
2	Beoordeling aanwezigheid van MEND-criteria<br>Herhaal de beoordeling van aanwezigheid van MEND-criteria conform beoordeling primaire stadiëring:<br>- Betrokkenheid van de MRF conform richtlijn, inclusief aan- of afwezigheid MEND-criterium (= minimaal 5 mm in diameter, willekeurig vlak);<br>- EMVI-aanwezigheid, en zo ja, gradering<br>- Aanwezigheid van tumordeposities (TD)<br>- Aanwezigheid van laterale lymfeklieren (LLN) ≥ 7 mm, en zo ja, links / rechts / beiderzijds met aantal<br>Benoem hierbij expliciet de respons van de MEND-criteria volgens volgende verdeling:<br>- Niet van toepassing (MEND-criterium op primaire MRI niet aanwezig)<br>- Complete respons<br>- Partiële respons<br>- Stabiel beeld<br>- Progressie en/of nieuw ontstaan van MEND-criterium
3	Mate van respons op basis van de tumor respons graad (TRG):<br>- T2W-MRI, volgens mrTRG<br>o TRG 5: Geen fibrose, tumor vergelijkbaar aanwezig (geen respons)<br>o TRG 4: Minder dan <25% fibrose, tumorsignaal dominant (enige respons)<br>o TRG 3: 50% tumor, 50% fibrose (matige respons)<br>o TRG 2: >75% fibrose, minimale intensiteit tumorsignaal (goede respons)<br>o TRG 1: Geen aanwijzingen voor tumorsignaal, of alleen minimale fibrose (complete respons)<br>- Respons op DWI<br>o 5: Tumor residu met zekerheid aanwezig<br>o 4: Tumor residu zeer waarschijnlijk<br>o 3: Tumor residu mogelijk, echter mogelijk een complete respons<br>o 2: Complete respons zeer waarschijnlijk<br>o 1: Duidelijk complete respons<br>- Algemene respons:<br>o Radiologisch complete respons: geen zichtbare tumorrest. Alleen littekenweefsel of fibrose. Geen restrictie op DWI.<br>o Near-complete response: subtiele restafwijking, twijfel tussen litteken en minimaal residu. Beperkte T2-afwijking, geen duidelijke restrictie.<br>o Goede response, niet complete: duidelijke resttumor zichtbaar, maar duidelijke volume afname.<br>o Stabiele ziekte: tumorafwijking is grotendeels onveranderd of minimaal afgenomen/toegenomen (<30% afgenomen, niet meer dan 20% toegenomen).

	○ Progressieve ziekte: tumor neemt onmiskenbaar toe (> 20% toegenomen), en/of er zijn nieuwe laesies ontstaan.

Voorstel tot radiologisch verslag restadiering na ICT/CRT inclusief aan- of afwezigheid MEND-criteria:
Tumor op [XX] mm vanaf de anorectale overgang.
Tumorrest | Litteken van [XX] tot [XX] uur.
Maximale lengte: [XX] mm.
MRF: niet betrokken | niet meer betrokken | betrokken, beperkt | betrokken: MEND-IT + (invasie).
EMVI: niet aanwezig | niet meer aanwezig | aanwezig (graad 4), wel goede respons | aanwezig (graad 4), onveranderd | aanwezig (graad 3), wel goede respons.

Respons:
Afname volume: circa 0-25% | 25-50% | 50-75% | >75%.
TRG: TRG 1, alleen fibrose complete remissie | TRG 2: grotendeels fibrose, minimale tumor resterend. | TRG 3: 50% tumorfibrose | TRG 4: <25% fibrose, grotendeels tumor | TRG 5: geen fibrose, geen respons.
DWI: 1, zeker complete respons | 2, waarschijnlijk complete respons | 3, mogelijk complete respons / mogelijk resttumor | 4, waarschijnlijk resttumor | 5, zeker resttumor.
Algemene indruk: radiologisch complete respons | near-complete respons | goede respons, zeker niet compleet | stabiele ziekte | progressieve ziekte.
T-Stadium: T1 | T2 | T1/T2 | T3a | T3b | T3ab | T3c | T3d | T4 (visceraal) | T4 (peritoneaal).

Lymfklieren:
Geen | 1 | 2 | 3 | 4 | 5 | 6 | 7 verdachte klieren resterend
Vasculaire deposities: nee | ja | mogelijk
Laterale klieren: nee | links | rechts | beiderzijds
N-stadium: N0 | N+

Conclusie:
Rectumcarcinoom, status na chemoradiatie | radiotherapie | neo-adjuvante chemotherapie.
Radiologisch complete respons | Near-complete respons | Goede respons, zeker niet compleet | Stabiele ziekte | Progressieve ziekte.
Voorstel tot stadiering: ycT 0 | 1 | 2 | 3a | 3b | 3ab | 3c | 3d | 4a | 4b N 0 | +, MRF - | +;beperkt | +;MEND-IT, EMVI - | + (graad 3) | + (graad 4), tumordeposities aanwezig | afwezig, [aantal] verdachte laterale klieren .

4. Contactgegevens

Voor vragen met betrekking tot dit protocol kunt u contact opnemen met:

Maaike Borg
Studiecoördinator MEND-IT
Catharina Ziekenhuis Eindhoven
E: mend-it@catharinaziekenhuis.nl
T: +31 (0)40 2396641

Voor vragen met betrekking tot de radiologische beoordeling kunt u contact opnemen met:

Dr. Joost Nederend
Radioloog
Catharina Ziekenhuis Eindhoven
E: joost.nederend@catharinaziekenhuis.nl

Bronnen:

Federatie Medisch Specialisten. Locoregionale stadiëring van rectumcarcinoom. In: Richtlijn Colorectaalcarcinoom. 2021. Beschikbaar op: https://richtlijnendatabase.nl/richtlijn/colorectaal_carcinoom_crc/diagnostiek_bij_crc/locoregionale_stadi_ring_rectumcarcinoom.html

Smith NJ, Barbachano Y, Norman AR, Swift RI, Abulafi AM, Brown G. Prognostic significance of magnetic resonance imaging-detected extramural vascular invasion in rectal cancer. Br J Surg. 2008 Feb;95(2):229-36. doi: 10.1002/bjs.5917. PMID: 17932879.
